# A Novel BA Complex Network Model on Color Template Matching

**DOI:** 10.1155/2014/918453

**Published:** 2014-08-19

**Authors:** Risheng Han, Shigen Shen, Guangxue Yue, Hui Ding

**Affiliations:** ^1^Nanhu College, Jiaxing University, Jiaxing 314001, China; ^2^College of Mathematics, Physics and Information Engineering, Jiaxing University, Jiaxing 314001, China

## Abstract

A novel BA complex network model of color space is proposed based on two fundamental rules of BA scale-free network model: growth and preferential attachment. The scale-free characteristic of color space is discovered by analyzing evolving process of template's color distribution. And then the template's BA complex network model can be used to select important color pixels which have much larger effects than other color pixels in matching process. The proposed BA complex network model of color space can be easily integrated into many traditional template matching algorithms, such as SSD based matching and SAD based matching. Experiments show the performance of color template matching results can be improved based on the proposed algorithm. To the best of our knowledge, this is the first study about how to model the color space of images using a proper complex network model and apply the complex network model to template matching.

## 1. Introduction

Template matching is an important technique in many application fields of computer vision, such as image registration, visual tracking, and recognition. There have been many template matching methods in the field of computer vision [1–5]. Recently, affine template matching methods [6–8] are attracting more and more attention. However, most studies of template matching algorithms focus on the gray images matching and there is still lack of a guidance theory to model the template matching process. In color image processing, many different color histograms are used in image indexing or image retrieval tasks [9–14]. For example, color indexing and color image query are proposed in [9, 10]. Color constant indexing methods which establish the histogram of color ratios are proposed in [11, 12]. Cumulative color histogram and fuzzy color histogram which are robust against illumination changes are proposed in [13, 14], respectively. Although the color histograms can be used as template features, it should be noted that the spatial information of the template's pixels is very important for template matching under conditions of affine transformation.

In fact, the color template matching is quite different from the gray template matching. For example, we found the traditional matching algorithm SAD (sum of absolute differences) based matching or SSD (sum of the squared differences) based matching cannot directly be used in the color image matching. Even the SAD or SSD similarity function has been transformed into RGB vector similarity, matching results of color images are still worse than the matching results of gray images. This is a quite strange phenomenon because color images matching should have been more robust than gray images matching.

Many studies have shown that the template matching process is easily affected by a variety of factors, such as image noise, similar blocks in background, and affine changing. In color space, the search space of matching actually becomes much larger than that in gray space. And these interference factors would also be amplified in the process of finding small parts of color images which match a color template.

To overcome these deficiencies, a novel BA complex network model of color space is proposed in this paper. The template's BA complex network model in color space can be used to select important color pixels which have much larger effects than other color pixels in matching process. At the same time, similar blocks interference and clutter background interference can also be suppressed based on the proposed BA complex network model. The proposed matching algorithm can be implemented by minimizing SSD or SAD. And experiments show the performance of color template matching results can be significantly improved based on the proposed algorithm.

In this paper, both the BA complex network model and color space are presented in Section 2. In Section 3, we present how to model the template as a BA complex network by using the template's color distribution, and then we describe how to select important color pixels which have much larger effects than other color pixels in matching process. Then a novel matching algorithm framework is proposed based on the BA complex network model of color space.  Section 4 presents the matching results under conditions of clutter background, similar blocks, and scale variation. At last, we present the conclusion and more extensive methods are advised.

## 2. A Brief Review on BA Complex Network Model and Color Space

In the real world, all kinds of networks exist everywhere, such as roads networks, biological networks, Internet, and telecommunication network. Many real world systems can be viewed as a collection of nodes that are linked to each other. Complex network theory is a powerful tool to model those complex systems and find statistical properties that characterize the structure of those networks. In addition, a proper complex network model can also predict complex system's behavior based on network evolution theory [15].

Recently, some study works have shown the complex network theory can be applied successfully in the field of image processing. In [16, 17], complex networks have been used in model texts, image textures, and face images. Shape descriptors based on a small-world network model are proposed in [18, 19]. However, there is still no related research about how to model color space of images using a proper complex network model. Next let us give a brief review on the BA complex network model which is related to our work.

### 2.1. BA Complex Network Model

Generally, a complex network model can be represented by a graph. A graph is a set of items, which are called vertices or nodes, with connections among them, called edges. A system taking the form of graph is also called a network. Let us give the definition of network as follows:

(1) $$\begin{array}{c} G=\left(N\left(G\right),\varepsilon \left(G\right)\right), \end{array}$$

where *N*(*G*) is vertices set, *ε*(*G*) is edges set, and the degree of vertex *i* is defined as being the number of edges which are bound to *i*, so the degree is usually denoted as *k*_*i*_. For an undirected network, *k*_*i*_ can be computed as follows:

(2) $$\begin{array}{c} k_{i}=\underset{j}{\sum }e_{i,j}, \end{array}$$

where *e*_*i*,*j*_ = {*i*, *j*}, *i*, *j* ∈ *N*(*G*), and *e*_*i*,*j*_ ∈ *ε*(*G*). In detail, *e*_*i*,*j*_ = {*i*, *j*} represents an edge between vertex *i* and vertex *j*. In graph theory, the degree of vertex *i* is usually denoted as *k*_*i*_. So *k*_*i*_ actually means the number of edges which are connected to the vertex *i*. From another angle, the *k*_*i*_ can also be regarded as the weight of the vertex *i*. For the connectivity of a network, the greater the value of *k*_*i*_, the greater the weight of the vertex *i*.

The BA complex network model is based on two generic mechanisms [20]: (i) growth of network: an evolving BA network continuously expands through the addition of new nodes and new links between nodes. (ii) Preferential attachment: more popular vertices of a network attract more new vertices in a BA network. A network model based on these two ingredients will reproduce the observed stationary scale-free distribution of degrees. The evolving process of BA complex network model can be described as follows.

(i) Growth of network: starting from a network which has *m*_0_ nodes, a new node *j* is added to the network, at each time step *t* = 1,2, 3,…, *M*.
(ii) Preferential attachment: the probability that a link will connect the new node *j* to an existing node *i* is proportional to the actual degree of *i*. And the probability that the new node *j* connects with *i* can be computed as follows:
  (3) $$\begin{array}{c} \underset{j\to i}{\prod }=\frac{k_{i}}{\sum_{l}k_{l}}. \end{array}$$

According to the BA complex network model, it should be noted that formula (3) is actually the generating rule of a BA complex network model. The effect of formula (3) is to choose a proper existing node *i* and let the new node *j* connect with it according to the probability of node *i*. As a result, these heavily linked nodes tend to get more links, while other nodes with only a few links are unlikely to be chosen as the destination for a new link. And then, the BA complex network model is sure to become a kind of scale-free networks.

The BA model has attracted much attention in the field of complex network. In addition to analytic and numerical studies of the model itself, many authors have proposed modifications and generalizations to make the model a more realistic representation of real networks [16, 21, 22]. Although all these kinds of networks are different in methods of the preferential attachment, they have one common point which is that their degree distributions follow power law. And all these networks can be called scale-free networks. The power law degree distribution of a network can be defined as follows:

(4) $$\begin{array}{c} p\left(k\right)\propto \alpha \cdot k^{-\gamma }, \end{array}$$

where both *α* and *γ* are constant parameters and *k* denotes the degree of each node in the network. According to formula (4), the power law distribution's function curve always has a heavy tail. All these networks whose degree distributions obey the power law are called scale-free networks.

In the study field of complex network, a simple way to quickly test whether a network obeys a power law distribution is to plot the network's degree distribution function on a log-log scales graph, which uses logarithmic scales on both the horizontal and vertical axes. Let us give the logarithm form of the power law distribution as follows:

(5) $$\begin{array}{c} \log\left(\alpha \cdot k^{-\gamma }\right)=-\gamma \log\left(k\right)+\log\left(\alpha \right). \end{array}$$

It is obvious that the power law distributions of BA complex network model and other scale-free networks should appear as straight lines in their logarithm forms.

Another important character of the BA network or other scale-free networks is that the connectivity is highly robust against random failures, because only those vertices, whose degrees are very large, have significant effects on the scale-free network [21]. This character is very helpful to a robust template matching algorithm or any other computer vision algorithms. In this paper, a new preferential attachment based on color histogram is proposed.

### 2.2. Color Space Model of Image

For color image processing, a pixel's color is a three-dimensional vector which can quantitatively describe a particular color value of the pixel. Then a color image can be defined as an *M* × *N* × 3 array of color pixels, where each color pixel is a three-dimensional vector.

#### 2.2.1. Pixel's Representation in RGB Color Space

There are many color spaces, such as RGB color space, YCbCr color space, and HSV color space. We choose RGB color space to build the BA network model because of the generality of RGB color space. Let us denote a pixel's color vector as *C*(*x*, *y*) in an image's RGB color space:

(6) $$\begin{array}{c} C\left(x,y\right)=\left[c_{\text{R}}\left(x,y\right),c_{\text{G}}\left(x,y\right),c_{\text{B}}\left(x,y\right)\right]. \end{array}$$

Then for an image of size *M* × *N*, there are *M* · *N* such color vectors which can be denoted as *C*(*x*, *y*), for *x* = 0,1, 2,…, *M* − 1 and *y* = 0,1, 2,…, *N* − 1.

According to (6), it can be seen that the color template matching is quite different from the gray template matching. Template's similarity function should be transformed into RGB vector similarity.

Different from other template matching algorithms, the proposed matching algorithm does not transform color template from color image to gray image. In Section 3, the proposed color template matching process will be carried out directly using the RGB vector. For keeping the consistency of formula symbol, the symbol *C*(*x*, *y*) or *C*(*x*_*t*_, *y*_*t*_) represents a RGB vector in the position of (*x*, *y*) or (*x*_*t*_, *y*_*t*_), respectively.

#### 2.2.2. Image's Color Distribution in RGB Color Space

In an image's color space, the color distribution can be represented by a color histogram of the image. The color histogram represents the number of pixels that have colors in each of a fixed number of color ranges. The color histogram can be produced from image's RGB color space.

Different from histogram of gray image, the color histogram is produced by discretization of colors in three channels (R, G, B). In the color histogram, the color image is transformed into a number of bins by counting the number of color pixels in each bin. So the color histogram of RGB color space is a three-dimensional histogram, and the size of the color histogram can be determined by the number of different RGB bins, such as 8 × 8 × 8 bins histogram and 16 × 16 × 16 bins histogram. For the convenience of analyzing, the three-dimensional histogram is transformed into one-dimensional in this paper. And this transformation is very important to reveal the scale-free characteristic of color images.

## 3. A Color Template Matching Algorithm Based on the BA Model of Color Space

In this paper, the template image region is regarded as a network, and the color distribution is viewed as a degree distribution of the network; then the network's degree distribution can be easily represented by the histogram of the color template.

### 3.1. The BA Complex Network Model Based on the Template's Color Distribution

Generally, an image's histogram represents the number of pixels at each different intensity values found in the image. In detail, the histogram is made up of bins; each bin represents a certain intensity range and the final value of each bin is the number of pixels assigned to it. A general histogram equation is shown as follows:

(7) $$\begin{array}{c} \prod \left({\text{Pixel}}_{j}⟶{\text{Bin}}_{i}\right)=\frac{{\text{Bin}}_{i}}{\sum_{u}{\text{Bin}}_{u}}. \end{array}$$

From (7), we can find the consistency between the histogram and BA complex network model which is defined in formula (3).

In theory, when we regard the template image region as a BA complex network, we can use (7) as the generating rules of the network. Because the definition form of (7) is consistent with (3), we called the template's model the template's BA complex network model.

It should be noted that the BA complex network model is a kind of simulation algorithm to produce complex network which has the scale-free characteristic. But (7) can be computed based on real image data. We do not use the BA complex network model to represent the geometric shape of the template, because the BA complex network model is only about topological relationship. In our BA complex network model, different pixels' color values are regarded as nodes, and the template's color histogram determines degree of each color value. So the proposed template's BA complex network model is also about topological relationship of color values. We will give the topological graph of the template's BA complex network model in Figure 3.

Based on the consistency between the histogram and BA complex network model, we want to reveal the scale-free characteristic of the template image region.

For example, when an image's color depth is 8 bits, there will be 2^24^ kinds of different color values in the RGB color space. However, for any real color image captured from the real world, there are far less than 2^24^ kinds of different color values in that image. Moreover, there are even a smaller number of color values occupying a large number in that image. We found that this phenomenon is not a coincidence. In most cases, the image histogram's bins are unbalanced, which means some pixels only occupy a small number in the template image. At the same time, a few pixels which have certain pixel value range, occupy a large number in the template image. And this phenomenon is also quite consistent with the BA scale-free network's definition.

In this paper, the template's one-dimensional color histogram is denoted as follows:

(8) $$\begin{array}{c} {\vec{Q}}_{\text{RGB}}={\left[q_{u}\right]}_{u=1,\ldots ,m}. \end{array}$$

Based on the definition of the BA complex network model and (7), the evolving process can be implemented according to the growth of color bins from *m* = 2^9^ = 8 × 8 × 8 to *m* = 2^24^ = 256 × 256 × 256. In each evolving step, the bin's number grows in the power of 2. Then the power law degree distribution can be achieved by sorting the color histogram in descending order as follows:

(9) $$\begin{array}{c} \text{Sort}\_\text{Descending}\left({\vec{Q}}_{\text{RGB}}\right)={\left[q_{s}\right]}_{s=1,\ldots ,m}, \end{array}$$

where each *s* is each bin's index of ${\vec{Q}}_{\text{RGB}}$ which has been sorted in descending order.

**Figure 1:**
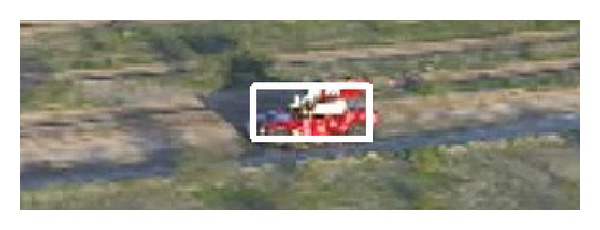
A template which is selected by a white rectangular.

At last, the power law distribution can be plotted on a log-log scales graph, which uses logarithmic scales on both the horizontal and vertical axes. Then let us give an example of a template's BA complex network model and the BA complex network's growth process. In Figure 1, a color template is selected by a white rectangular.

**Figure 2:**
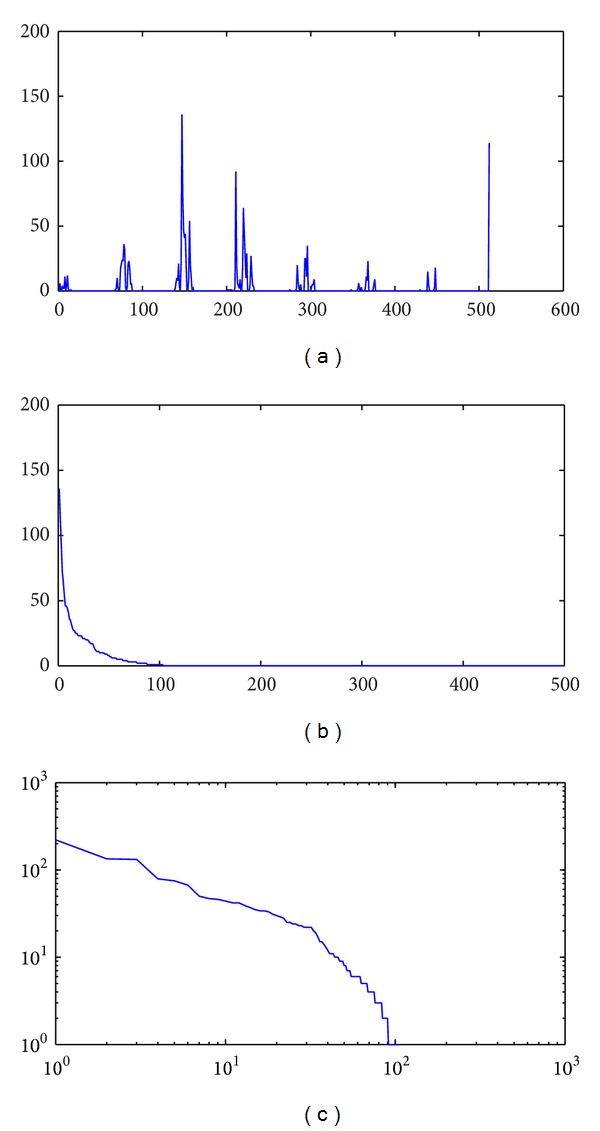
Three different forms of the template's color histogram (512 bins).

Then the template's color histogram (8 × 8 × 8 bins) which has been transformed into one-dimensional (2^9^ = 512 bins) is shown in Figure 2(a).

The template's color histogram is sorted in descending order and the sorted histogram is shown in Figure 2(b). It is obvious that the sorted histogram can be fitted by power law distribution. For revealing the scale-free characteristic, the sorted histogram is plotted in log-log scales, and the logarithm form is shown in Figure 2(c).

**Figure 3:**
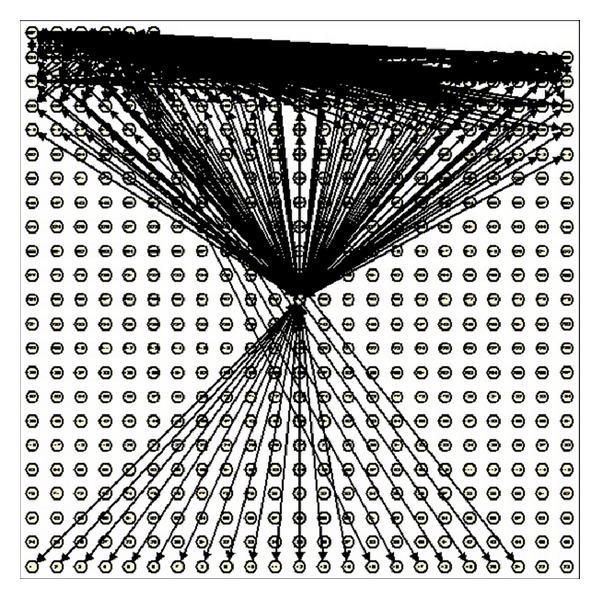
The topological graph of the template's BA complex network model.

According to our BA complex network model's definition, the network's degree distribution is represented by the histogram of the color template. According to the template's histogram in Figure 2(a), there should be 512 nodes in the network's topological graph. In addition, the color histogram determines degree of each node, and then we can produce the network's topological graph in Figure 3.

In Figure 3, there are three kinds of nodes. Firstly, these nodes without edges mean there are no these colors in the template. Secondly, those nodes which have edges mean that those colors exist in the template. Thirdly, a small number of nodes have most edges. The third kind of nodes represents those important pixels in the template. It is clear that only limited colors exist in the template, and there are even a smaller number of colors values occupying a large number in the template. Figure 3 shows the proposed network model has very strong scale-free characteristic.

According to the growth character of BA complex network model, the histogram's evolving process is carried out according to the growth of color bins from *m* = 2^9^ = (8 × 8 × 8) to *m* = 2^21^ = (128 × 128 × 128). Then, the evolving process of the template's color histograms is shown as follows.

**Figure 4:**
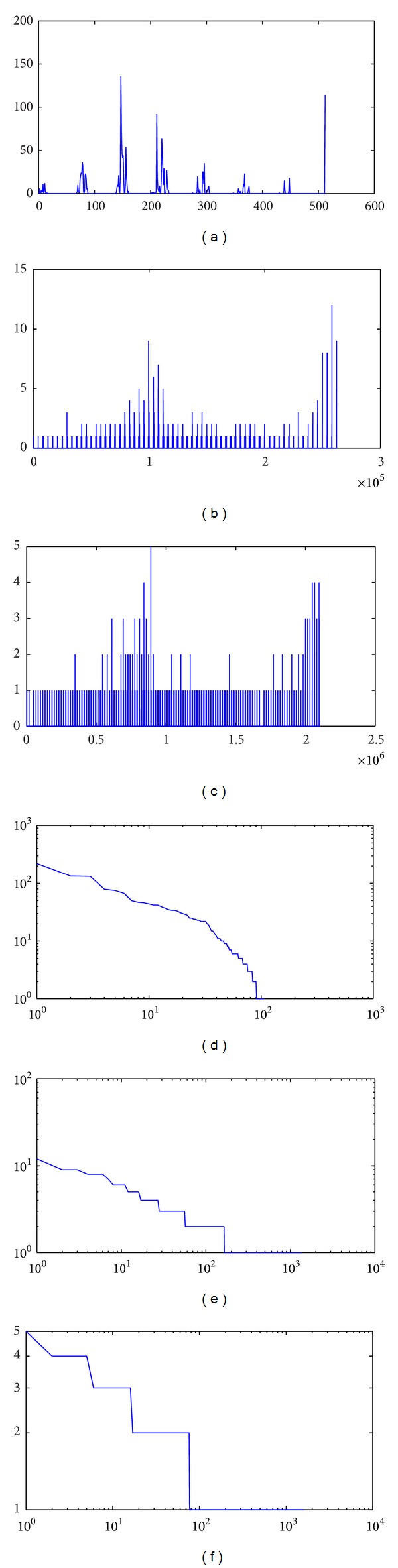
The evolving process of the template's color histograms (from 8 × 8 × 8 = 512 bins to 128 × 128 × 128 = 2097152 bins).

In Figures 4(a)–4(c), the template's color histograms are plotted with 8 × 8 × 8 bins, 64 × 64 × 64 bins, and 128 × 128 × 128 bins, respectively. And then, these histograms' logarithm forms, after they have been sorted in descending order, are shown in Figures 4(d)–4(f). Because the BA complex network model's degree distribution should appear as an approximation of a straight line in log-log scales, the shape change of Figures 4(d)–4(f) shows that the scale-free characteristic becomes more and more apparent.

In addition, this evolving process can also be carried out more precisely from bins' number: *m* = 2^9^ = 8 × 8 × 8 to *m* = 2^24^ = (256 × 256 × 256). In each evolving step, the bin's number grows in the power of 2. Because our algorithm simply uses the color histogram as BA complex network model and mainly makes use of the BA complex network's scale-fee characteristic, there is no need to draw and analyze the networks topological graph in matching process. This modeling method is very important to reduce the computation cost.

Moreover, by making use of the scale-free characteristic, the number of colors of the template image can be reduced by only choosing those important color pixels which have larger effects than other color pixels in matching process. Next, let us introduce the proposed method in detail.

### 3.2. Template's Important Pixels Based on the BA Complex Network Model

From the definition of the BA complex network model and above examples, the scale-free characteristic of color space has been revealed. Then the template's BA complex network model of color space can be used to select important color pixels which have much larger effects than other color pixels in matching process. Based on (9), let us give the definition of BA template as follows:

(10) BA_Template={〈xt,yt〉∈Template ∧C(xt,yt)≥Threshold ∧Threshold=argmins(TopN(qs)) ∧TopN(qs)=Rounding(γ·BINSR·BINSG·BINSB)}.

In (10), (*x*_*t*_, *y*_*t*_) is the pixel's position of the template and *C*(*x*_*t*_, *y*_*t*_) is the pixel's color value at the position (*x*_*t*_, *y*_*t*_). The top *N* values of the sorted color histogram are selected into the template's BA network model, so the Threshold can be used for adjusting the network's size. More importantly, the BA network's evolving mechanism is also integrated by using the number of Bins in RGB color space. *γ* is a proportion parameter to determine how many bins of histogram are most important. Because *γ* ∈ [0,1], a rounding function is used. The *q*_*s*_ has been defined in (9). The symbol ∧ is a logical operator which is called Conjunction or And. In short, (10) is a set of pixels whose importance is much larger than others, and the set is called BA_Template.

In the stage of matching, the image being searched should also be transformed into a bigger BA network model using the following equation:

(11) $$\begin{array}{c} \text{BA}\_\text{IM}=\left\{\left(x_{i},y_{i}\right)\in \text{IM}\wedge C\left(x_{i},y_{i}\right)\geq \text{Threshold}\right\}. \end{array}$$

In (11), IM is the search image which will be matched by template. According to (11), only those image's pixels which have high similarity to template can be kept by using the same threshold parameter of (10). Before using the BA_Template and BA_IM into the template matching process, the BA_IM need to be filtered as follows:

(12) F_IM={C(xi,yi) ∣ ((xi,yi)∈BA_IM∧C(xi,yi)=0)   ⟶C(xi,yi)=−255}.

The purpose of filtering is to eliminate inferences which come from unimportant pixels and noise in the matching process.

### 3.3. Matching Algorithm Based on the BA Template in Color Space

By using (10) and (12), the matching process can be implemented only based on those important pixels. For matching in color space, the traditional matching algorithms should be altered firstly. And then the proposed BA_Template and F_IM can be integrated into the matching process. The proposed color template matching process can be defined as a process of minimizing SSD (sum of the squared differences), and the minimizing process can be carried out in color space as follows:

(13) (x,y)match  =argmin(xi,yi)(SSD(F_IM(xi,yi),BA_Template(xt,yt))).

In (13), (*x*,*y*)_match_ is the matching result, and the SSD minimizing process in RGB color space can be formulated as follows:

(14) SSD(F_IM(xi,yi),BA_Template(xt,yt))  =∑xt=−Sx Sx∑yt=−SySy((F_IMR(xi+xt,yi+yt)−  BA_TemplateR(xt,yt))2+(F_IMG(xi+xt,yi+yt)−BA_TemplateG(xt,yt))2+(F_IMB(xi+xt,yi+yt)−  BA_TemplateB(xt,yt))2),

where *S*_*x*_ and *S*_*y*_ are the scales of the template in *x* and *y* directions, respectively. And the subscripts R, G, B represent the three color components of the BA_Template and the F_IM in RGB color space.

Then let us give the description of the proposed color template matching algorithm as follows. 

*Algorithm*. Color template matching based on BA model.

*Input*. Input images which include the template and another search image. 

*Step 1*. Determine a template region: Template = Rect (*C*_*t*0_, *S*_*x*_, *S*_*y*_, BINS, *γ*), where *C*_*t*0_ is the center of the selected template *S*_*x*_ and *S*_*y*_ are the scales of the template in *x* and *y* directions, BINS is the number of bins of the Template's color histogram, and *γ* is the parameter used in (10). 

*Step 2*. Compute the color histogram of the Template region and transform the three-dimensional color histogram into one-dimensional histogram like the definition of (8). 

*Step 3*. Determine the Template's power law according to (9). 

*Step 4*. Produce the BA_Template according to (10). 

*Step 5*. Produce the search image's BA_IM and F_IM according to (11) and (12). 

*Step 6*. Matching by minimizing the SSD according to (13) and (14). 

*Output*. The matching position *X*_match_ which is found in the search image.

In the proposed algorithm, the color template is regarded as a BA network model, and its color distribution is regarded as the degree of the BA network model. Based on the BA network's robust character, those important color pixels are selected into the BA_Template. And then the search image is also processed properly by selecting and filtering. At last, the matching process is implemented by minimizing the SSD between BA_IM and F_IM.

## 4. Experimental Results

The color template matching experiments are carried out under conditions of clutter background, scale change, and similar color inference.

**Figure 5:**
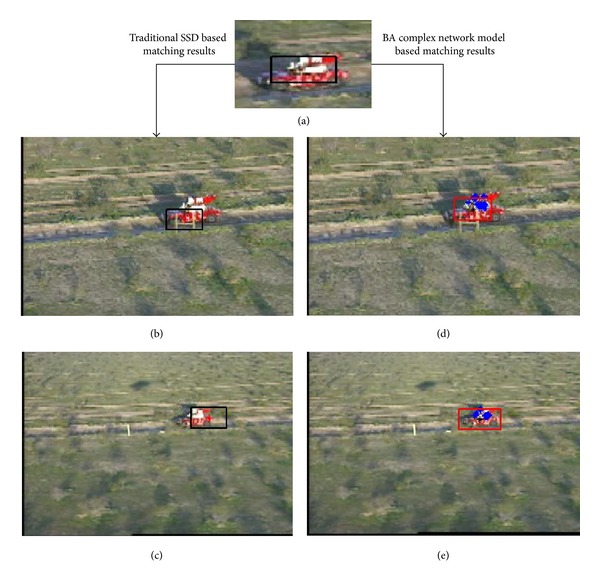
Matching results compared under conditions of partial occlusion, scale change, and clutter background.

In the first experiment, the template is selected from one image and matched to another image; the proposed matching algorithm will find the selected template under conditions of shadow inference, partial occlusion, and clutter background. In the second experiment, the matching experiment is implemented under the condition of scale change. Both experiments are shown in Figure 5.

**Figure 6:**
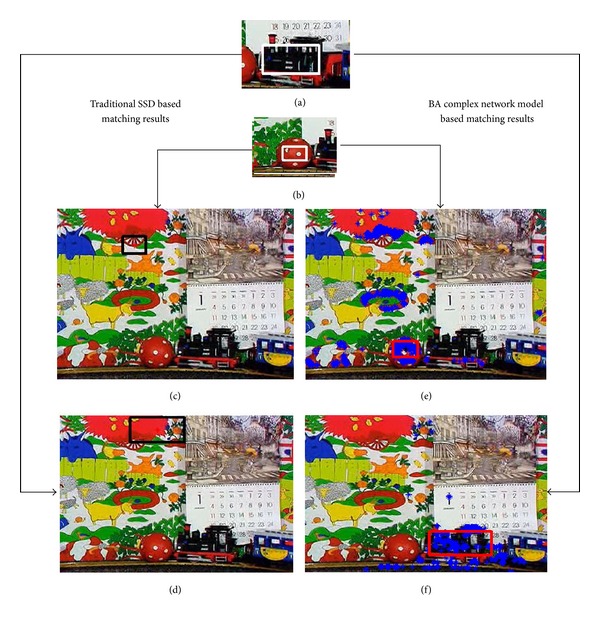
Matching results compared under severe conditions of similar color's interference and complex background.

In the third and the fourth experiments, the matching experiments are carried out in a more severe environment where there are many image blocks which are very similar to the template's color. In addition, the template's positions are also changed in the search image. Both the third and the fourth experiments are shown in Figure 6. All our matching results are compared to traditional SSD based matching results.

The SSD is one of the most popular similarity metrics in the field of template matching, and the matching process can be defined as looking for the minimum score of SSD. For the matching result, the smaller the SSD score value is the better. So, we list the SSD scores of our all matching experiments in Tables 1 and 2, respectively.

Firstly, let us give the first and the second experiments in Figure 5.

(a) The template is selected by a black rectangular.
(b) Matching result of traditional SSD under condition of partial occlusion.
(c) Matching result of traditional SSD when the target's scale changes.
(d) Matching result of the proposed algorithm under condition of partial occlusion.
(e) Matching result of the proposed algorithm when the target's scale changes.

In Figure 5, those blue color points are important special points, which are determined by the template's BA complex network model. The color template is shown in Figure 5(a). According to Figures 5(b) and 5(d), we can see that both the traditional SSD matching algorithm and the proposed algorithm can find the template's position although a slight partial occlusion exists, but the result of the proposed algorithm appears a little more robustly and precisely.

Figure 5(c) shows that matching result of the traditional SSD algorithm is getting worse, when the target's scale changes. Figure 5(e) clearly shows that the proposed matching algorithm can be carried out successfully under conditions of scale change.

It is easy to see that the proposed algorithm is more robust against the partial occlusion and scale change than the traditional SSD based matching algorithm.

By using our template's BA complex network model, we can see that the proposed matching algorithm can get smaller SSD score values than the traditional SSD based matching algorithm.

Then, let us give the third and the fourth experiments in Figure 6. Different to Figure 5, there are two different templates which are shown in Figures 6(a) and 6(b).

In addition, those blue points also mean labels of important color pixels in Figure 6.

(a) The first template is a toy train which is selected by a white rectangular.
(b) The second template is a part of red ball which is selected by a white rectangular.
(c) The red ball's matching result of traditional SSD under conditions of similar color.
(d) The toy train's matching result of traditional SSD in complex background.
(e) The red ball's matching result of the proposed algorithm under conditions of similar color.
(f) The toy train's matching result of the proposed algorithm in complex background.

Because there are many image blocks which are very similar to the template's color in Figure 6, the matching conditions of the third and the fourth experiments are very severe and challenging for most template matching algorithms. Figures 6(c) and 6(d) show the traditional SSD based matching algorithm completely fails to match the target. Figures 6(e) and 6(f) show that the proposed matching algorithm can be carried out successfully under conditions of similar color interference and complex background.

In these matching experiments, because there is much similar color's interference, SSD scores become smaller. In this situation, on one hand, the template's BA complex network model can enhance the template by selecting those important pixels. On the other hand, by transforming and filtering the search image, inference can be suppressed in the matching process. As a result, the proposed algorithm can get lower SSD scores than the traditional SSD based matching algorithm.

## 5. Conclusion and Discussion

There are two major contributions in this paper. Firstly, a novel BA complex network model of color space is proposed. Then the BA model's evolving process is implemented according to the growth of color bins. Secondly, the proposed algorithm can select template's important color pixels which have much larger effect than other pixels based on the BA model's scale-free characteristic. The proposed matching algorithm is robust against random interferences, such as clutter background, scale change, and similar color inference.

Because of the generality of the template's BA model, many further extensions and improvements can be made. For example, the template's BA model can also be used as a tracking template which only includes important pixels. Based on the definition of template's BA model of color space, many other traditional matching methods (such as SAD based matching and NCC based matching) can be integrated into the proposed algorithm easily. In conclusion, this paper demonstrates the potential of applying BA complex network theory to color template matching problem.

## Figures and Tables

**Table 1:** SSD scores in the first and the second experiments.

Matching experiments	Scores of traditional SSD based matching results	Scores of BA complex network model based matching results
The first experiment	7.6667	6.0518
The second experiment	8.6678	6.4512

**Table 2:** SSD scores in the red ball matching and the toy train matching experiments.

Matching experiments	Scores of traditional SSD based matching results	Scores of BA complex network model based matching results
The red ball matching experiment	4.5042	3.6667
The toy train matching experiment	4.3378	0.3031
